# Exploring the protective mechanisms of the yunpi jiedu tongluo qushi decoction on methotrexate-induced reproductive damage in male rats based on Nrf2/HO-1 signaling pathway

**DOI:** 10.3389/fcell.2025.1626955

**Published:** 2025-08-22

**Authors:** Wu Chen, Min Chen, Dian-Ming Li, Wei-Man Shi, Min Zhang, Zhi-Yu Li, Lin Huang, Cheng-Ping Wen, Qiao Wang

**Affiliations:** ^1^ Zhejiang Chinese Medicine University, Hangzhou, Zhejiang, China; ^2^ Key Laboratory of Chinese Medicine Rheumatology of Zhejiang Province, Hangzhou, China; ^3^ Department of Nephrology, The First Affiliated Hospital of Zhejiang Chinese Medical University (Zhejiang Provincial Hospital of Chinese Medicine), Hangzhou, China

**Keywords:** yunpi jiedu tongluo qushi decoction, methotrexate, oxidative stress, cell apoptosis, reproductive damage

## Abstract

**Background:**

Methotrexate (MTX), a folate antagonist, is widely used in the treatment of tumors and autoimmune diseases such as rheumatoid arthritis (RA), due to its antiproliferative, antimetabolic, and potent anti-inflammatory properties. However, high-dose or long-term use of MTX can result in toxicity to the gastrointestinal, hemopoietic, and reproductive tissues. Yunpi Jiedu Tongluo Qushi Decoction (YJT), an effective Chinese herbal formula, is utilized in RA treatment to mitigate the toxic and side effect of MTX. Despite its clinical application, the precise effects and the underlying mechanisms through which it mitigates reproductive damage have yet to be elucidated.

**Purpose:**

This study was conducted to investigate the protective effects and mechanisms of YJT against MTX-induced reproductive toxicity.

**Methods:**

Here we used an integrated approach combining Ultra Performance Liquid Chromatography-Mass Spectrometry (UPLC-MS), *in vitro* and vivo experiments, and molecular docking technology to systematically elucidate the protective effects and mechanism of YJT.

**Results:**

We identified nine active ingredients in YJT that contributed to its efficacy. YJT alleviated testicular damage in MTX-exposed rats, resolved abnormalities of sperm morphology, structure, and quality, decreased the number of TUNEL-positive cells in testicular tissue, and reduced oxidative stress (OS). YJT affected GC2spd cell apoptosis by regulating OS response. Both *in vitro* and vivo experiments showed that YJT can activate the Nrf2/HO-1 signaling pathway against OS response. Molecular docking results further confirmed the strong binding activity between the core components of YJT and Nrf2, HO-1.

**Conclusion:**

Collectively, our findings indicate that YJT protects against MTX-induced apoptosis in spermatogenic cells by inhibiting OS and Nrf2/HO-1 signaling pathway. This study provides a theoretical foundation and experimental basis for the use of YJT to alleviate MTX-induced reproductive toxicity.

## Introduction

Methotrexate (MTX), a folate antagonist, was first introduced over 50 years ago. Since then, it has been widely used to treat malignancies and autoimmune diseases like rheumatoid arthritis (RA), psoriasis, psoriatic arthritis, and inflammatory bowel disease (Marin et al., 2022; Sukhotnik et al., 2013). Current guidelines from the European League Against Rheumatism (EULAR) and the American College of Rheumatology (ACR) recommend MTX as a first-line therapy for RA (Sukhotnik et al., 2013; Naewla et al., 2023). This is probably due to its antiproliferative, antimetabolite, and potent anti-inflammatory effects. However, despite its clinical benefits, MTX is often associated with potential adverse effects, However, despite its clinical benefits, MTX is associated with significant adverse effects including gastrointestinal injury, hepatotoxicity, nephrotoxicity, bone marrow suppression, pulmonary toxicity, and reproductive toxicity (Wani et al., 2023; Manna et al., 2023). MTX can impair spermatogenesis and reduce sperm motility in males (Mansour et al., 2021). In the male reproductive system, spermatogenic cells undergo rapid proliferation and division, rendering them highly vulnerable to toxins (Zhou et al., 2023). Exposure to cytotoxic substances such as MTX and copper can inhibit spermatogenic cell proliferation and induce cell death. Guo H’s study showed (Guo et al., 2021) that overexposure causes reproductive toxicity and induces testicular and sperm damage. Prolonged MTX exposure may also impair ovarian function and diminish reproductive potential in women (Brouwer et al., 2013). The detailed mechanism behind this effect has not yet been fully elucidated. Emerging research suggests that oxidative stress and apoptotic cell death contribute to MTX-induced toxicities, including hepatotoxicity and nephrotoxicity (Mishra et al., 2023). Hussein OE et al. demonstrated (Hussein et al., 2020) that prolonged exposure to high levels of MTX can induce reactive oxygen species (ROS)-mediated inflammation, cell apoptosis, and oxidative stress (OS) in mouse liver. Therefore, further research is needed to investigate the role of ROS and apoptosis in reproductive damage caused by MTX.

Traditional Chinese medicine (TCM) is a significant complementary medical approach. It has been proven effective and promising in the treatment of RA. The search for therapeutic agents from natural Chinese herbs to mitigate inflammation and toxicity offers a promising strategy (Guo et al., 2021; Chen, 2021; Grosen et al., 2017). YJT is a formula developed by Prof. Cheng-ping Wen, based on the principles of “activating the spleen, detoxifying the body, eliminating dampness, and promoting collateral circulation”. It is composed of Atractylodes macrocephala, Coicis Semen, Angelicae Sinensis Radix, Lonicerae Japonicae Flos, Polygoni Cuspidati Rhizoma Et Radix, Scolopendra, Paeoniae Radix Alba, Cuscutae Semen, Glycyrrhizae Radix Et Rhizoma Praeparata Cum Melle. Noteworthy, previous studies have found that the combination of YJT and MTX can improve symptoms such as joint swelling and pain, reduce CRP, ESR, and other indicators, and decrease the relapse rate in RA patients (Chen, 2021). However, the efficacy of YJT in alleviating MTX-induced reproductive damage has not yet been investigated. Relevant reports suggested that the constituents of Coicis Semen in YJT are effective in suppressing and ameliorating reproductive impairment (Wang et al., 2022; Han et al., 2023), such as Polydatin in Smilax glabra can attenuate lipid peroxidation damage in mouse testicular tissue cells and effectively inhibit the DNA damage of testicular cells by OS (Ma and Jia, 2018). Coicis Semen can upregulate the levels of serum testosterone in androgen-deficient rats, ameliorate the abnormalities of sexual organs, and increase seminal plasma fructose content, thus improving sperm activity and reproductive capacity in rats. Based on these findings, we suggest that YJT alleviates MTX-induced reproductive damage by reducing OS and preventing cell apoptosis. Therefore, this study is designed to explore the *in vivo* reproductive toxicity of MTX, with a focus on the role of OS and apoptosis. Additionally, we aim to investigate the detoxifying effect and specific mechanism of YJT in alleviating reproductive damage both *in vivo* and *in vitro*.

## Materials and methods

### Materials and reagents

MTX was procured from Beijing Solabo Technology Co., Ltd. (Beijing, China). Primary antibodies and secondary antibodies against Bcl2, Bax, cleaved-Caspase3, Nrf2, and HO-1 were acquired from Hangzhou Huaan Biotechnology Co., Ltd. (Hangzhou, China). Reactive oxygen species detection kits were supplied by Beyotime Biological Technology Co., Ltd. (Shanghai, China). Dulbecco’s Modified Eagle Medium (DMEM) was obtained from Gibco (Carlsbad, California, United States). Fetal bovine serum (FBS) was sourced from Gibco (Carlsbad, California, USA). Glutathione (GSH), superoxide dismutase (SOD), and malondialdehyde (MDA) were purchased from Nanjing Jiancheng Bioengineering Institute (Nanjing, China). Annexin V-FITC/PI double staining apoptosis analysis kits, RIPA lysis buffer, tetramethyl azide salt, and PMSF were obtained from Shanghai Beyotime Biological Technology Co., Ltd (Shanghai, China). Reverse transcription kits were procured from Nanjing Novozymes Biological Technology Co., Ltd. (Nanjing, China).

### The preparation of traditional Chinese medicine decoction

The composition of the YJT includes Atractylodes macrocephala (Cang zhu, 15 g), Coicis Semen (Chao Yi Yi Ren, 15 g), Angelicae Sinensis Radix (Dang gui, 10 g), Lonicerae Japonicae Flos (Jin Yin Hua, 15 g), Polygoni Cuspidati Rhizoma Et Radix (Hu Zhang, 15 g), Scolopendra (Wu Gong, 1 piece), Paeoniae Radix Alba (Chao Bai Shao, 9 g), Cuscutae Semen (Tu Si Zi, 12 g), and Glycyrrhizae Radix Et Rhizoma Praeparata (Zhi Gan Cao, 6 g). YJT has traditionally been administered in clinical practice in the form of a decoction. To remain consistent with clinical application, this method was prioritized to accurately reflect the formulation used by patients. The YJT extract (Lot No. 191205) was provided by Sichuan Xin Green Pharmaceutical Technology Development Co., Ltd. YJT was infused in warm water to obtain concentrations of 100 mg/mL and 150 mg/mL.

### Ultra-high performance liquid chromatograph (UPLC-MS) analysis

For quality control of YJT, UPLC-MS analysis was conducted using a Welch AQ-C18, 150 × 2.1 mm, 1.8 µm column on an UltiMate 3000 RS UPLC-MS system (Thermo Fisher Scientific, Massachusetts, United States). The separation was carried out using a gradient elution with a mobile phase consisting of 0.1% formic acid in water (A) and methanol (B) (0 min: 2% B, 5 min: 20% B, 10 min: 50% B, 15 min: 80% B, 20 min: 95% B, 27 min: 95% B, 28 min: 2% B, 30 min: 2% B). The flow rate was set at 0.3 mL/min, and the column temperature was maintained at 32 °C. The injection volume was 5.00 µL.

To further confirm the reliability of HPLC, we conducted HPLC-MS analysis. Mass spectrometry was performed on Thermo Orbitrap Exploris (New York, United States). The analytical column was the AQ-C18,Welch (150 mm × 2.1 mm, 1.9 μM) column maintained at a column temperature of 35 °C. The mobile phase comprised Methanol (B) and an aqueous solution of 0.1% formic acid (A). The gradient elution profile was as follows: from 0 to 5 min, the composition changed from 2% to 20% B; from 5 to 10 min, it increased from 20% to 50% B; then from 10 to 15 min, it rose from 50% to 80% B; then from 15 to 20 min, it rose from 80% to 95% B; then keep the gradient at 95% B for 3 min and finally, from 27 to 30 min, it transitioned completely to 2% B. The flow rate was established at a rate of 0.3 mL/min with an injection volume set at 5 μL. The electrospray ion (ESI) source adopted the positive ion and negative ion scanning modes. The conditions used for the ESI source were as follows: the positive and negative ion voltages are both 23.2 kV, respectively. The temperature of the ion transfer tube is 325 °C, the vaporization temperature is 350 °C, the sheath gas flow rate is 40Arb, the auxiliary gas flow rate is 15Arb, and the purge gas flow rate is 0Arb.

### Animals and drug administration

This protocol received approval from the Animal Research Committee of Zhejiang Chinese Medical University and adhered to the guidelines established in the Guidance for the Care and Use of Laboratory Animals (Permission number: 20210809-10). Male Wistar rats aged 7-8 weeks, were procured from Shanghai Bikai KeYi Technology Co., Ltd (Shanghai, China). Their body weights ranged from 200 to 250 g. The license for the use of experimental animals was issued under the number SYXK (Zhe) 2021-0,012. The animals were housed at 25 °C ± 1 °C and, 50% ± 5% humidity with a 12-h light/dark cycle and had ad libitum access to food and water. The rats were randomly divided into five groups: the control group (n = 10), MTX group (n = 10), To establish a model of MTX-induced reproductive injury, rats in all experimental groups (excluding controls) received intraperitoneal injections of MTX ( 20 mg/kg) once daily for 5 consecutive days starting on day 7. After adaptive feeding, at a dose of 20 mg/kg/day (Sukhotnik et al., 2013; Hussein et al., 2020; Mahmoud et al., 2020; Armagan et al., 2008; Mahmoud et al., 2017), except for those in the control group. In the medium-dose YJT group (YJT + M), YJT drug suspension was administered orally at a dose of 50 mg/mL/kg per day. In the high-dose YJT group (YJT + H), YJT drug suspension was administered orally at a dose of 100 mg/mL/kg per day. In the FA group, FA drug suspension was administered orally at a dose of 5 mg/kg per day. The control group received drinking water using the same method of administration. YJT was carried out via the intragastric route for 28 consecutive days. In previous studies, the 28-day intervention implemented by YJT has been shown to improve serum inflammatory marker levels and alleviate pain-related indicators in animal models (such as rats and mice), with good safety profiles (Wang et al., 2024; Lou et al., 2022; Jiang et al., 2024). Therefore, this 28-day regimen was also selected for the reproductive toxicity study of this formulation. On day 35, rats were anesthetized by intraperitoneal injection of tilramine-zoletil (Zoletil 100®, Virbac S.A, France) at a dose of 1 mg/kg body weight (equivalent to 0.01 mL of reconstituted solution per kilogram of body weight, containing 100 mg/mL of active ingredient). Deep anesthesia was confirmed by observing the disappearance of toe reflexes. After collecting cardiac blood, rats were euthanized by intraperitoneal injection of 50 mg/kg sodium pentobarbital (Sinopharm Chemical Reagent Co., Ltd., China). After death was confirmed, testes, epididymides, and sperm were collected for subsequent analysis.

### Evaluation of serum sex hormone levels

After obtaining blood samples by cardiac puncture., centrifugation was carried out at 4 °C and 5,000 revolutions per minute (rpm) for 10 min, and the supernatant was collected. Subsequently, the serum was separated and used to detect the sex hormone levels. The levels of sex hormones were evaluated by the serum levels of Testosterone(T) Luteinizing Hormone (LH) and Follicle-Stimulating Hormone (FSH) using enzyme-linked immunosorbent assay (ELISA) kits (Ruixin Biotechnology Co., Ltd. Quanzhou, China) following the manufactures’ instructions, respectively.

### Detection of sperm quality

The distal portion of the left epididymis was placed in 2 mL of pre-warmed DMEM culture medium containing 5% FBS at 37 °C. Following mincing, the tissue was thoroughly homogenized to yield a sperm suspension, which was then allowed to left undisturbed for 1 min. Next, 200 μL of this suspension was added into 2 mL of fresh, pre-warmed DMEM medium (containing 5% FBS) at 37 °C, and the mixture was incubated within a thermostatically controlled water bath for 5 min. A 10 μL sample of the suspension was dispensed onto a hemocytometer to account sperm concentration (expressed as 10^6 cells per milliliter). A 20 μL sample from this preparation was transferred onto a pre-warmed microscope slide for evaluating sperm motility under a light microscope utilizing ×400 magnification. The sperm motility, activity percentages and abnormality rate were calculated according to the method outlined in Table 1 (Cosentino et al., 1985).

**TABLE 1:** Spermatogenesis grading system (Johnsen’s score).

Score	Characteristics
10	Complete spermatogenesis with many spermatozoa present
9	Slightly impaired spermatogenesis with many late spermatids, disorganized epithelium
8	Less than five spermatozoa per tubule, few late spermatids
7	No spermatozoa, no late spermatids, many early spermatids
6	No spermatozoa, no late spermatids, few early spermatids
5	No spermatozoa or spermatids, many spermatocytes
4	No spermatozoa or spermatids, few spermatocytes
3	Spermatogonia only
2	No germinal cells, Sertoli cells only
1	No seminiferous epithelium

### Determination of the coefficient of rats’ reproductive organs

At the end of the treatment or recovery phase, blood was harvested from all rats prior to their humane euthanasia. Thereafter, a macroscopic examination of the testes and epididymis was conducted to assess their overall appearance. The weights of testes and epididymis were recorded and organ coefficients were calculated independently.

### Histopathological evaluations

The left testicular tissues obtained from rats were immersed in 4% paraformaldehyde solution for fixation. Post-fixation, tissues underwent a series of rinses in 70% ethanol with repeated changes, followed by dehydration, clarification, and infiltration with paraffin wax for embedding. These paraffin-embedded tissues were sectioned into 5-μm thick slices, which were then affixed to glass slides, subjected to deparaffinization, rehydration, and stained with hematoxylin and eosin (H&E) stains. Slides were subsequently sealed with neutral balsam. Pathological morphology of the rat testicular tissues was visualized using a digital slide scanning system (Hamamatsu, Japan). These measurements were conducted by evaluating transverse sections of 30 seminiferous tubules across five fields of view. Thirty circular or near-circular tubules were selected from three testis sections of each animal (Mu et al., 2023; Oufi and Al-Shawi, 2014). Examination was conducted at ×100 magnification under a light microscope. Histological assessment of changes in the germinal series cells was performed based on the following criteria: sloughing (clusters of spermatocytes sloughing off from the epithelium of the seminiferous tubules), exfoliation (clusters of germ cells released into the lumen of the seminiferous tubules), and vacuolization (formation of cavities within the seminiferous tubules) (Orazizadeh et al., 2014). According to Cosentino’s score (Cosentino et al., 1985) (Table 2) and for spermatogenesis according to Johnsen’s scoring system (Johnsen, 1970) (Table 1).

**TABLE 2:** Histopathological grading of seminiferous tubules (Cosentino’s score).

Grade	Characteristics
I	Normal testicular architecture with an orderly arrangement of germinal cells
II	Injury showed less orderly, non-cohesive germinal cells and closely packed seminiferous tubules
III	Injury exhibited disordered sloughed germinal cells, with reduced size of pyknotic nuclei and less distinct seminiferous tubule borders
IV	Injury exhibited seminiferous tubules that were closely packed with coagulative necrosis of the germinal cells

### Apoptosis analyses by TUNEL method

Following the sectioning of rat testicular tissues, standard protocols for deparaffinization and rehydration were executed. Thereafter, the In situ Apoptosis Detection kit (TaKaRa Biotechnology Co., Ltd., Kyoto, Japan) was employed for apoptotic cell detection. Following deparaffinization and hydration, proteinase K digestion was carried out at ambient temperature for 15 min. The tissue sections were then rinsed with 1×PBS. Subsequently, Terminal Deoxynucleotidyl Transferase (TdT) enzyme was added, and the reaction was conducted at 37 °C for 60 min. Afterward, the sections were incubated with anti-fluorescein antibody at 37 °C for 30 min. After adding the reaction solution, sections underwent hematoxylin staining, alcohol gradient dehydration, and were sealed with neutral resin. The cells-stained brown was regarded as positive apoptotic cells by TdT-mediated dUTP Nick-End Labeling (TUNEL) staining and apoptosis levels were observed by light microscopy.

### Cell culture

The GC-2spd cells (GuanDao, 20211016-58) were obtained from Shanghai Guandao Biotechnology Co., Ltd. (Shanghai, China). GC-2spd cells were cultured in Dulbecco’s Modified Eagle Medium (DMEM) containing 10% fetal bovine serum, 50 U/mL penicillin, and 50 μg/mL streptomycin. The culture conditions were maintained at 37 °C, in a 5% CO_2_ atmosphere, and at a relative humidity of 85%–95%. Subsequent experiments were conducted when the cell confluence reached 80%–90%.

### Cell viability assay

The GC-2spd cells were seeded in a 96-well culture plate at a density of 5 × 10^3 cells per well and were incubated overnight at 37 °C in a humidified chamber containing 5% CO2. Following incubation, the cells were treated with various concentrations of MTX (0, 0.4, 0.8, 1.6, 2.0, 2.4, 3.2 μg/mL) for either 24 h or 48 h. After the respective treatment duration, 10 μL of the Cell Counting Kit-8 (CCK-8) reagent was added to each well. The absorbance was then measured at 450 nm, and the cell viability was calculated accordingly. A similar method was used to evaluate the effects of hydrogen peroxide (50-400 mol/L) and YJT (12.5-800 μg/mL) on the viability of GC-2spd cells.

### Apoptosis analyses by flow cytometry

Cell apoptosis was assessed by FITC/PI double staining and flow cytometry analysis. GC-2spd cells were cultured in a 6-well plate for 12 h, pre-treated with MTX (0.4,0.8,1.6 μg/mL) for 24 h, and then exposed to different concentrations of YJT for 24 h (Supplementary Figure S2D–F). The impact of YJT on cell apoptosis was evaluated using an Annexin V-FITC/PI detection kit, and the effects of H_2_O_2_ on the viability of GC-2spd cells were determined using the same method.

### Detection of OS indices

The levels of MDA, GSH, and SOD are critical parameters reflecting the potential antioxidant capacity. Subsequently, the content of MDA, SOD, and GSH was determined using the following colorimetric assay kits (Nanjing Jiancheng Bioengineering Institute, Nanjing, China).

### Detection of ROS

For *in vivo* experiments, cryostat sections of the testicular (thickness = 10 μm) were sliced and incubated with dihydroethidium (Sevier Institute of Biotechnology, Wuhan, China). Dihydroethidium was oxidized by ROS to fluorescent ethidium, which binds to nucleic acids to create a bright-red fluorescence in the nucleus. The bright-red fluorescence from DHE was detected using a fluorescence microscope Observer. A1 (Zeiss, Oberkochen, Germany). For *in vitro* experiments, GC-2spd cells were pre-treated with 200 μmol/L H_2_O_2_ for 24 h, followed by treatment with various concentrations of YJT for 24 h. The levels of ROS were measured using the ROS Assay Kit (Beyotime of Biotechnology, Shanghai, China).

### Quantitative real-time PCR (RT-qPCR) analysis

Total RNA was extracted using Trizol Reagent (Takara, Kusatsu, Japan). The extracted total RNA was reverse transcribed into cDNA using the Rever Tra Ace qPCR RT Kit (Toyobo, Osaka, Japan) in a T100TM Thermal Cycler (Bio-Rad, CA, United States). Subsequently, the cDNA was utilized for RT-qPCR analysis with Ultra SYBR Mixture (cwbiotech, Jiangsu, China) in a Light Cycler 96 (Roche, Basel, Switzerland). The primer sequences for Bcl-2, Bax, Caspase-3, Nrf2, GSH, SOD, HO-1, CAT, and GAPDH were listed in Tables 3, 4. The 2^−△△CT^ method was employed to calculate the relative mRNA expression levels.

**TABLE 3:** Primers for rat qRT-PCR.

Gene	forward (5′-3′)	reverse (5′-3')
Nrf2	TTGTAGATGACCATGAGTCGC	TGTCCTGCTGTATGCTGCTT
GSH	GGGTCGCTCTTTAGGGCTTT	TTGCATCGAAGGTCCTCCAC
SOD	ACACAAGGCTGTACCACTGC	CCACATTGCCCAGGTCTCC
HO-1	GTAAATGCAGTGTTGGCCCC	ATGTGCCAGGCATCTCCTTC
CAT	CGACCGAGGGATTCCAGATG	ATCCGGGTCTTCCTGTGCAA
Bax	AAGAAGCTGAGCGAGTGTCT	CAAAGATGGTCACTGTCTGC
Bcl2	CGCCCGCTGTGCACCGAGA	CACAATCCTCCCCCAGTTCACC
GAPDH	CAGGCATATGGGTGGTCCATAG	TCATGGGATCCACCTGCAGC

Nrf2, Nuclear factor E2-related factor 2, GSH, Glutathione; SOD, Superoxide Dismutase; HO-1, Heme oxygenase-1; CAT, Catalase; Bax, BCL2-associated X protein, Bcl2, B-cell lymphoma-2, GAPDH, Glyceraldehyde-3-phosphate dehydrogenase.

**TABLE 4:** Primers for mouse qRT-PCR.

Gene	Forward (5′-3′)	Reverse (5′-3')
Nrf2	CAGCCATGACTGATTTAAGCAG	CAGCTGCTTGTTTTCGGTATTA
Bax	TTGCCCTCTTCTACTTTGCTAG	CCATGATGGTTCTGATCAGCTC
Bcl2	GATGACTTCTCTCGTCGCTAC	GAACTCAAAGAAGGCCACAATC
SOD	TGTCCATTGAAGATCGTGTGAT	TCATCTTGTTTCTCATGGACCA
CAT	TCCCTGCTGTCTCACGTTCCG	GCTGCTCCTTCCACTGCTTCATC
HO-1	CACCTTCAAGTTGGTTAATGCA	CATGACCTGGATGTAAAACGTC
GAPDH	GGCAAATTCAACGGCACAGTCAAG	TCGCTCCTGGAAGATGGTGATGG

Nrf2, Nuclear factor E2-related factor 2, Bax, BCL2-associated X protein, Bcl2, B-cell lymphoma-2, SOD, Superoxide Dismutase; CAT, Catalase; HO-1, Heme oxygenase-1; GAPDH, Glyceraldehyde-3-phosphate dehydrogenase.

### Western blotting

Cell total protein was extracted on ice for 30 min using RIPA lysis buffer containing PMSF (1:100). Subsequently, the protein was loaded onto a 12% sodium dodecyl sulfate-polyacrylamide gel electrophoresis (SDS-PAGE) and transferred to a polyvinylidene fluoride (PVDF) membrane. The membrane was then blocked with 5% BSA and incubated with the primary antibody overnight at 4 °C. After washing the PVDF membrane the next day, it was incubated with the secondary antibody at room temperature for 2 h. Following another round of washing, the protein bands were visualized using the Odyssey Li-COR CLx infrared imaging system (LI-COR, Nebraska, United States), and the grayscale values of the protein bands were analyzed using ImageJ software.

### Molecular docking

Molecular docking was conducted to confirm the binding affinity of the selected core components to the key targets. The target protein’s 3D crystal structure was obtained from the RCSB database (http://www.rcsb.org/pdb) (Goodsell et al., 2020),while the 3D structures of the compound ligands were obtained from PubChem. We utilized Open Babel 3.1.1 (http://openbabel.org/wiki/Main_Page) to convert the format of the ligand-receptor files to pdb format ((O'Boyle et al., 2011). Autodocking Tools 1.5.6 was then applied to the basic procedure, including receptor dehydration and hydrogenation, energy minimization of the chemical synthesis distributor, ligand atom type assignment, and Gasteiger charge calculations. Finally, we saved the file in pdbqt format. After that, molecular docking was performed using Autodocking Vina 1.1.2 to obtain binding energy values, and the combinations with the lowest binding energy were visualized and analyzed by PyMOL 2.4.0 software. A negative binding energy value indicates a thermodynamically spontaneous binding process between the ligand and target protein. A more negative value (i.e., lower binding energy) correlates with higher binding affinity and greater conformational stability of the complex. In this study, a binding energy value of <-7 kcal/mol was used as a threshold to corroborate the credibility of the action relationship between the active ingredient and the core target. Finally, we constructed a heat map of the ligand-receptor binding energy using GraphPad Prism 9.0.

### Statistical analysis

Statistical analysis was performed using SPSS 26.0 and GraphPad Prism 9.0. Continuous data are presented as mean ± standard error (SEM). For normally distributed data, one-way analysis of variance (ANOVA) was utilized. In cases where the data did not follow a normal distribution, non-parametric tests were employed. A significance level of *P* < 0.05 was considered statistically significant.

## Results

### The quality control of YJT

**FIGURE 1:**
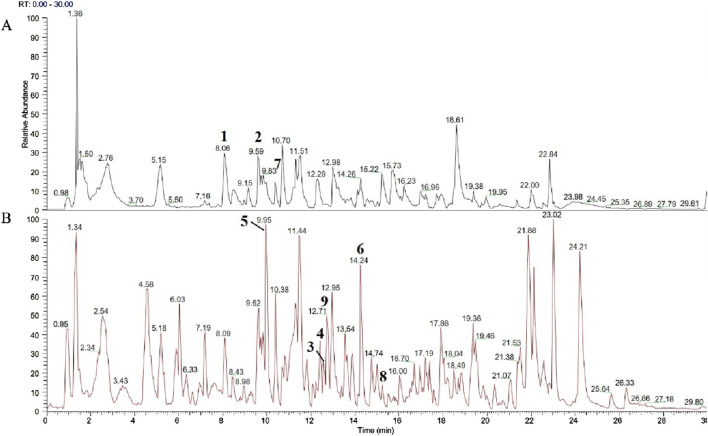
Analysis of YJT by UPLC-MS. The chromatographic peaks in the fingerprint are labeled as follows: **(A)** Black is the negative ion mode total ion flow diagram. Neochlorogenic acid (1), Chlorogenic acid (2), Caffeic acid (7) **(B)** Red is the positive ion mode total ion flow diagram. Cynaroside (3), Quercetin (4), Paeoniflorin (5), Benzoyl paeoniflorin (6), Caffeic acid (7), Emodin (8), Hyperoside (9).

The results of UPLC-MS analysis of YJT were illustrated in Figure 1; Supplementary Figure S1. We identified nine major compounds, including neochlorogenic acid in Atractylodes macrocephala, chlorogenic acid, and Cynaroside in Honeysuckle, quercetin and Hyperoside in Sophora japonica, paeoniflorin and benzyl paeoniflorin in Paeonia lactiflora, Caffeic acid in Angelica sinensis, and Emodin in Cuscuta vulgaris.

### Safety and effectiveness of YJT on methotrexate-induced reproductive damage in male rats

**FIGURE 2:**
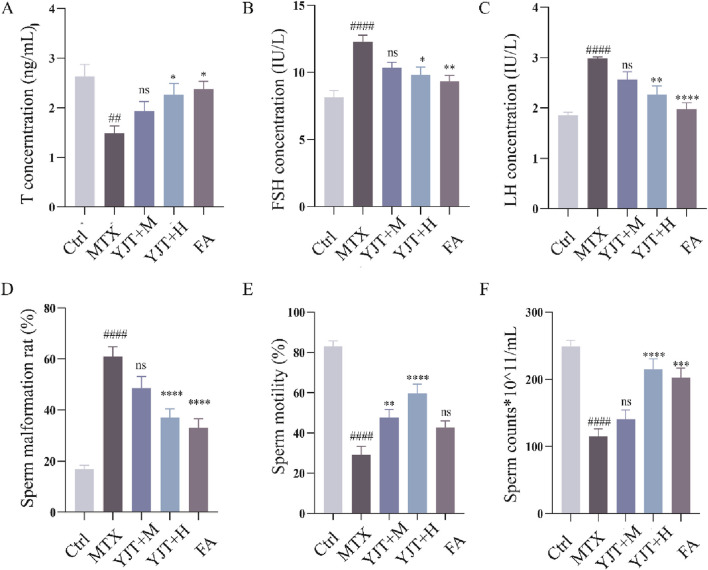
YJT effectively ameliorated MTX induced Male rats’ abnormality of serum hormone levels, malformation rate, density and viability. **(A–C)** Changes in serum T **(A)**, FSH **(B)**, and LH **(C)** levels in different rat groups (n = 6–10). **(D–F)** Abnormal sperm morphology (head defects, coiled tails, double tails, and tailless sperm were recorded as abnormal sperm), sperm density, and sperm motility in different rats groups (n = 6–10). Data are presented as Mean ± SEM. # Compared to the control group; * Compared to the MTX group; ns No significant difference; ##*P* < 0.01; ###*P* < 0.001; *P < 0.05; **P < 0.01; ***P < 0.001.

*In vivo* safety assessment of YJT was evaluated in MTX-induced rats by examining body weight and liver function. The results revealed that YJT intervention improved body weight loss in rats (*P* < 0.01, Supplementary Figure S2A) while having no significant effect on liver injury indices (Supplementary Figure S2B,C). In addition, we evaluated the efficacy of YJT using sex hormone levels, spermatozoa damage and testicular histopathological damage in rats. T synthesis is essential for germ cell development and maturation, and LH and FSH are involved in regulating and stimulating T synthesis. In the case of a decrease in serum T levels, a feedback mechanism is triggered, leading to an increased secretion of FSH and LH (Gökçe et al., 2011). As Figures 2A–C, MTX decreased T levels (*P* < 0.01), increased FSH (Figure 2B) and increased LH levels (*P* < 0.001), and When rats were treated with YJT at a concentration of 100 mg/mL, serum T levels increased, while FSH and LH expression levels decreased; however, these changes were not statistically significant compared to the MTX group. When treated with YJT at a concentration of 150 mg/mL, serum T, FSH, and LH levels all significantly recovered. Sperm morphology, density, and motility are fundamental indicators for assessing sperm fertility potential. Sperm malformation rate, density and motility are the fundamental indexes used to evaluate sperm fertility potential. We found that sperm malformation rate significantly increased (Figure 2D, *P* < 0.001), sperm density and sperm motility significantly decreased (Figures 2E,F, *P* < 0.001) in MTX-intervened rats. In contrast, Medium-dose YJT and high-dose YJT interventions significantly improved rat sperm motility, while the improvement in sperm motility after FA treatment was not statistically significant (*P* < 0.001, *P* < 0.01, *P* > 0.05). Additionally, High doses of YJT and FA significantly reduce sperm malformation rates and increase sperm counts, but the results of medium-dose YJT intervention are not statistically significant (*P* < 0.0001,*P* < 0.001, *P* > 0.05).

**FIGURE 3:**
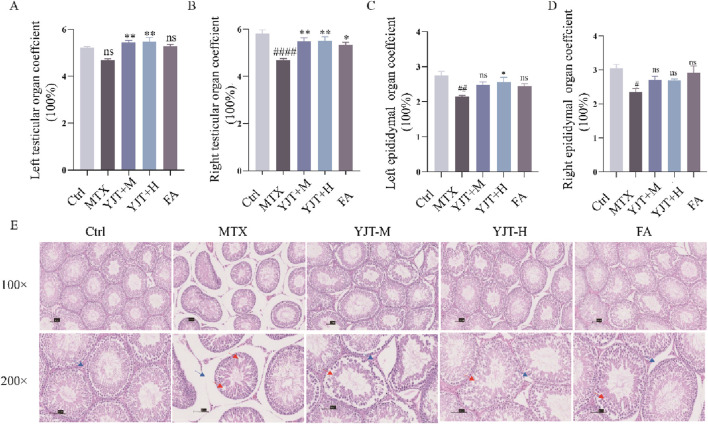
YJT effectively ameliorated MTX induced Male rats’ reproductive organs coefficients and histopathological changes. **(A,B)** Changes in the organ coefficients of the left and right testes in rats (n = 5–8). **(C,D)** Changes in the organ coefficients of the left and right epididymides in rats (n = 5–8). **(E)** Histological sections of testicular tissues from rat groups stained with hematoxylin and eosin. Representative images of testicular tissue sections are shown. Blue arrows represent interstitial cells, red arrows represent spermatogenic cells that are irregular, detached or surrounded by vacuolated tissue (original magnification ×40 and ×200). Data are presented as Mean ± SEM. # Compared to the control group; * Compared to the MTX group; ns No significant difference; #*P* < 0.05; ##*P* < 0.01; ###*P* < 0.001; *P < 0.05; **P < 0.01; ***P < 0.001.

Figures 3A–D described the impact of MTX on testicular and epididymal organ coefficients. Following MTX intervention, both the testicular organ coefficient and epididymal organ coefficient in rats showed a decreasing trend, with statistical significance observed in all cases except for the left testicular organ coefficients (*P* < 0.001, *P* < 0.01, *P* < 0.05, *P* > 0.05). Following YJT intervention, the organ coefficients of the testes and left epididymis in rats significantly increased (*P* < 0.001, *P* < 0.01), with all changes showing statistical significance except for the right epididymis organ coefficients. While FA showed the same therapeutic effect (*P* < 0.05). As shown in Figure 3E, MTX induced disruption of the epithelial cell structure of the varicocele in rats, reduction of the germ cell hierarchy, reduction of sperm cells in the varicocele, irregularity in the morphology of the germ cells, as well as irregularity, detachment, and vacuolization of the surrounding tissues. And YJT and FA interventions significantly reduced intratesticular pathology.

### Effects of YJT on methotrexate-induced spermatogenic cell apoptosis

**FIGURE 4:**
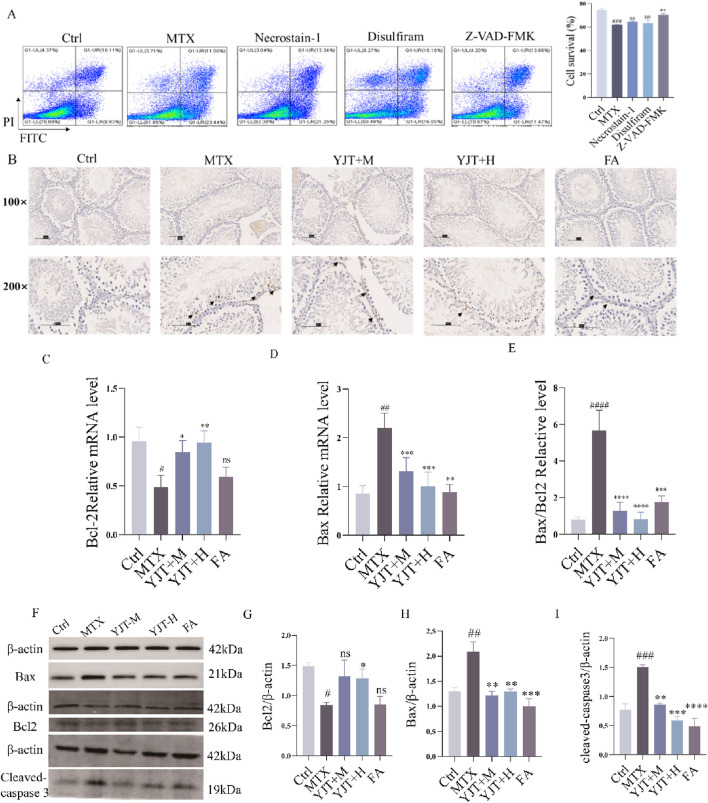
Effects of YJT on MTX-induced cell apoptosis. **(A)** Effects of various cell death inhibitors on MTX-induced apoptosis in spermatogenic cells (n = 3). **(B)** TUNEL immunohistochemical staining to detect apoptotic cells in testicular tissues from different rats groups (arrows). Representative images of testicular tissue sections are shown (original magnification ×100 and ×200). **(C–F)** mRNA expression levels of Bcl2 **(C)**, Bax **(D)**, and Bax/Bcl2 **(E)** in rats testicular tissues (n = 6–10). **(F–I)** Protein expression levels of Bcl2 **(G)**, Bax **(H)**, and cleaved-caspase 3 **(I)** in testicular tissues from various rats groups (n = 3). Data are presented as Mean ± SEM. # Compared to the control group; * Compared to the MTX group; ns No significant difference; #*P* < 0.05; ##*P* < 0.01; ###*P* < 0.001; *P < 0.05; **P < 0.01; ***P < 0.001.

To investigate the mechanism of MTX-induced spermatozoa damage. The flow cytometry was employed to explore the cell death pattern of germ cells (Figure 4A). Cell survival was significantly decreased after MTX intervention (*P* < 0.001). When we intervened with the caspase inhibitor Z-VAD-FMK, cell survival was increased (*P* < 0.01), whereas the necrosis inhibitor and cytolytic inhibitor had no effects on cell survival. The results described above indicated that apoptosis is the main mode of cell death in MTX-induced germ cell reduction.

To investigate the specific mechanism of germ cell apoptosis, we established a GC2spd cell injury model by using MTX (Supplementary Figure S3A–C). We found that MTX increased the mRNA expression level of Bax (*P* < 0.001), and decreased the mRNA expression level of Bcl2 (*P* < 0.001). YJT decreased the Bax mRNA expression and increased the mRNA expression of Bcl2 (*P* < 0.001), and this effect varied dose-dependently over a range. Similar to the *in vitro* experiments, we observed the same effect in the *in vivo* experiments. TUNEL staining showed that the control group had a few TUNEL-positive cells and the rats' testicular tissue appeared normal with no nuclear condensation or apoptotic bodies in germ cells (Figure 4B). MTX increased the number of TUNEL-positive cells, especially in spermatogonia, spermatocytes, and sperm cells. YJT showed a dose-dependent reduction in TUNEL-positive cells in testicular tissues and ameliorated MTX-induced apoptosis at the protein and mRNA levels (Figures 4C–I).

### OS mediated MTX-induced spermatogenic cell apoptosis and the role of YJT

**FIGURE 5:**
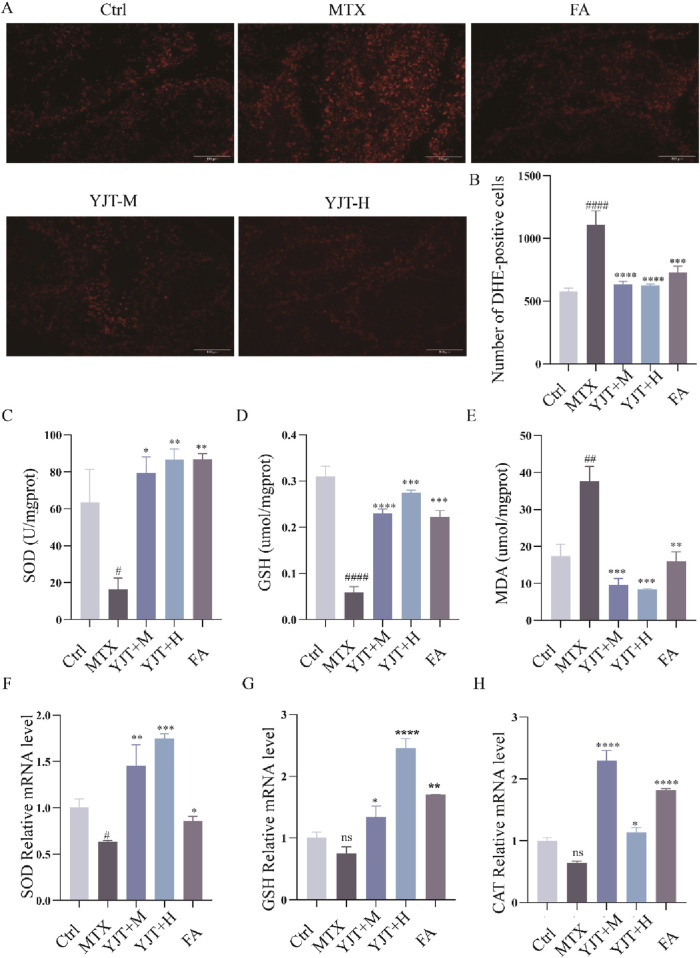
YJT improved oxidative damage in MTX-induced male rats’ testicular tissues. **(A,B)** Expression levels of ROS in testicular tissues of rats in various groups (n = 5–8). **(C–E)** Colorimetric assays to detect the expression levels of SOD **(C)**, GSH **(D)**, and MDA **(E)** in testicular tissues of rats in different groups s (n = 5–8). **(F–H)** mRNA expression levels of SOD **(F)**, GSH **(G)**, and CAT **(H)** in rats testicular tissues (n = 5–8). Data are presented as Mean ± SEM. # Compared to the control group; * Compared to the MTX group; ns No significant difference; #*P* < 0.05; ##*P* < 0.01; ###*P* < 0.001; *P < 0.05; **P < 0.01; ***P < 0.001.

**FIGURE 6:**
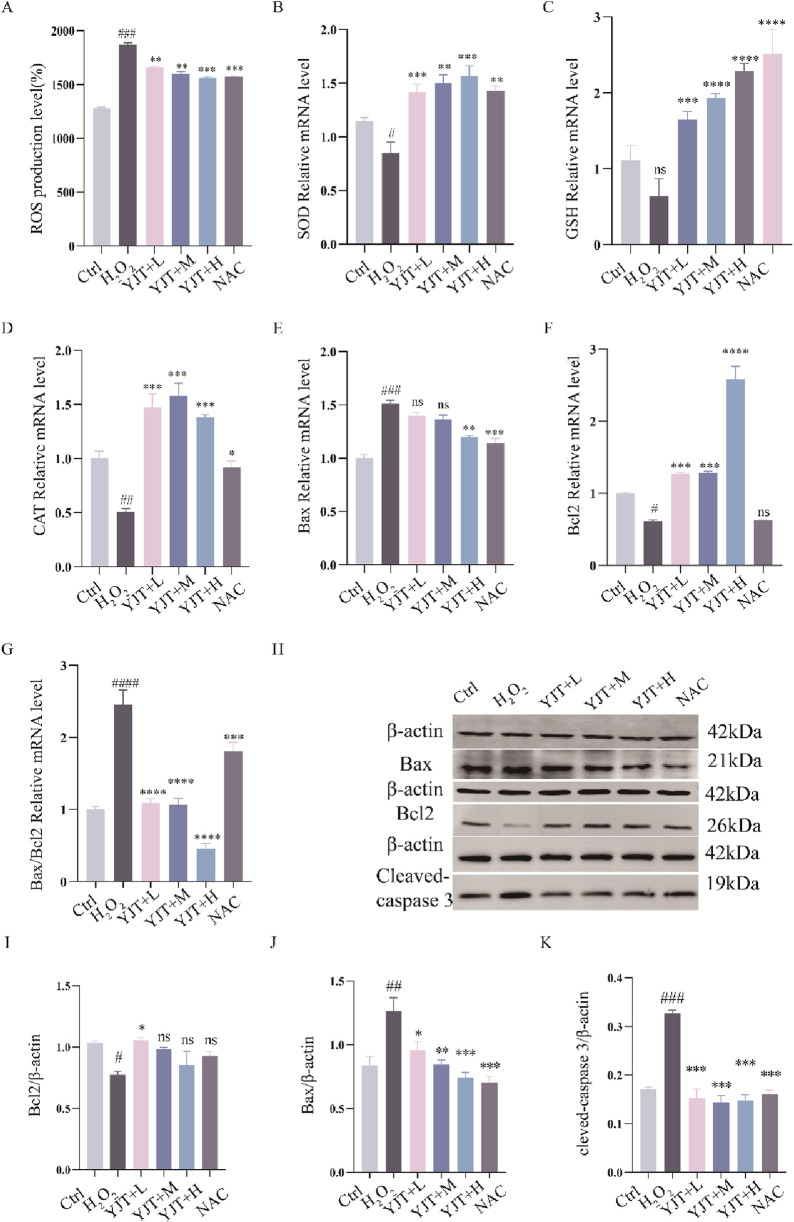
YJT improves oxidative damage in GC2spd cells. **(A)** Expression levels of ROS in the H_2_O_2_-induced oxidative damage model of GC2spd cells (n = 5–7). **(B–D)** Colorimetric assays to detect the expression levels of SOD **(B)**, GSH **(C)**, and CAT **(D)** in the H_2_O_2_-induced oxidative damage model of GC2spd cells (n = 9). **(E–G)** mRNA expression levels of Bax **(E)**, Bcl2 **(F)**, and Bax/Bcl2 **(G)** in the H_2_O_2_-induced oxidative damage model of GC2spd cells (n = 9). **(H–K)** Protein expression levels of Bcl2 **(I)**, Bax **(J)**, and cleaved-caspase3 **(K)** in the H_2_O_2_-induced oxidative damage model of GC2spd cells (n = 3). Data are presented as Mean ± SEM. # Compared to the control group; * Compared to the MTX group; ns No significant difference; #*P* < 0.05; ##*P* < 0.01; ###*P* < 0.001; *P < 0.05; **P < 0.01; ***P < 0.001.

We investigated the potential role of OS in MTX effects. As Figures 5A–E, MTX downregulated the protein levels of SOD and GSH and up-regulated the protein levels of ROS, MDA in rat testis (*P* < 0.001). YJT protected against oxidative damage by increasing the expression of SOD and GSH and decreasing the expression of ROS, MDA. And at the mRNA expression level, we also found that MTX down-regulated the levels of SOD, GSH and CAT mRNA expression in rat testis (*P* < 0.05, *P* < 0.01), and YJT significantly upregulated the expression levels of OS indicators and showed a dose-dependent pattern (Figures 5F,G). These results suggested that MTX-induced OS and apoptosis in germ cells, which were alleviated by YJT treatment through the rebalancing of antioxidant enzymes. To explore the role of OS in the apoptotic effects of germ cells, we established an oxidative damage model in spermatogenic cells using H_2_O_2_ (Supplementary Figure S4). The *in vitro* results showed that YJT was effectively reducing the oxidative damage of GC2pds as in the *in vivo* experiments (Figure 6A).

It has been found (Alsousi and Igwe, 2019) that OS is an essential pathway for inducing apoptosis, and the results of flow cytometry showed that the apoptosis rate in the oxidative damage model was significantly elevated (Supplementary Figures S3D,E, S5D,E). And MTX increased Bax and Bax/Bcl2 mRNA expression (*P* < 0.001) and decreased Bcl2 mRNA expression (*P* < 0.001), while apoptosis induced by OS was reversed by YJT treatment. Western blotting analysis likewise confirmed these findings (Figures 6H–K).

### Effects of YJT on Nrf2/HO-1 signaling pathway

**FIGURE 7:**
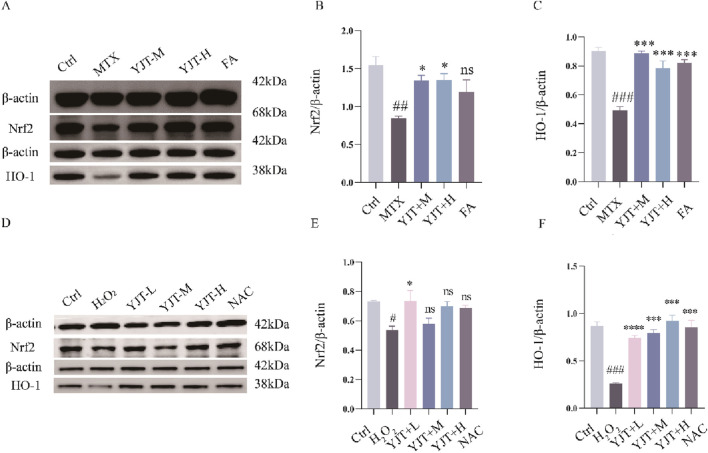
Effects of YJT on Nrf2/HO-1 signaling pathway. **(A–C)** The impact of Nrf2 and HO-1 protein expression levels in the testicular tissues of the different rats groups (n = 3). **(D–F)** The impact of Nrf2 and HO-1 protein expression levels in the H_2_O_2_-induced oxidative damage model of GC2spd cells (n = 3). Data are presented as Mean ± SEM. # compared to the control group; * compared to the MTX group; ns indicates no significant difference, #*P* < 0.05; ##*P* < 0.01; ###*P* < 0.001; *P < 0.05; **P < 0.01; ***P < 0.001.

This study investigated the impact of YJT on Nrf2, as a key transcription factor for cellular antioxidant defense. The role of YJT in MTX-induced reproductive damage was investigated through Western blot and RT-PCR analyses. As shown in Figures 7A–C, it was observed that YJT upregulated the expression levels of Nrf2 and HO-1 induced by MTX, and FA showed the same trend on the expression levels of Nrf2 and HO-1. These results were further confirmed in the H_2_O_2_-induced oxidative damage model of GC2spd cells, where YJT increased the expression of Nrf2 and its downstream target HO-1 at both the mRNA and protein levels in testicular tissues and GC2spd cells (Figures 7D–F; Supplementary Figure S5A–C).

### Molecular docking

**FIGURE 8:**
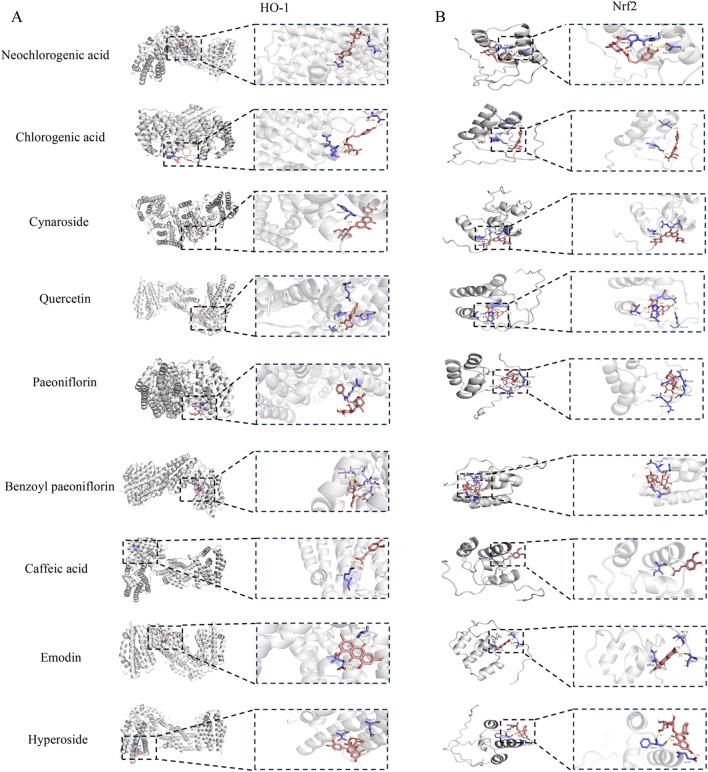
Molecular docking results. **(A)** Molecular docking pattern diagram. **(B)** Heat map of ligand-receptor binding energy.

We have identified two key targets, HO-1 and Nrf2, through preliminary experimental investigations. Additionally, UPLC-MS compositional analysis of YJT has successfully identified nine crucial components (Figure 1). We conducted molecular docking of these identified core targets with pivotal components. The docking results (Figure 8A) generated 18 sets of receptor-ligand pairs, with binding scores for potential targets ranging from −7 kcal/mol to −5 kcal/mol and an average binding energy of −7.01 kcal/mol, indicating a robust binding activity between the core components of YJT and the predicted key targets (Figure 8B; Table 5). Additionally, we found significant differences in binding affinity between Nrf2/HO-1 activators and the positive control drug FA (Supplementary Figure S6). Notably, FA exhibited moderate binding affinity for Nrf2(-6.5 kcal/mol), but unexpectedly strong affinity for HO-1 (−8.8 kcal/mol). In contrast, the positive control sulforaphane (Nrf2 activator) exhibited weak binding to Nrf2(-3.0 kcal/mol), while cobalt protoporphyrin IX (CoPPIX, HO-1 inducer) demonstrated high-affinity binding to HO-1 (−10.0 kcal/mol, Supplementary Figures S6, S7) (The Protein Data Bank (PDB) IDs of the proteins used in the docking study and the PubChem compound IDs (CIDs) of the ligands are shown in Supplementary Figure S7).

**TABLE 5:** Molecular docking results table of key active ingredients in YJT.

Active constituents	Key targets	
	HO-1	Nfr2
Neochlorogenic acid	−7.3	−5.8
Chlorogenic acid	−6.9	−5.9
Cynaroside	−8.3	−6.7
Quercetin	−8.3	−7.1
Paeoniflorin	−8.4	−6.2
Benzoylpaeoniflorin	−8.6	−6.8
Caffeic acid	−6.1	−4.7
Emodin	−8.1	−6.1
Hyperoside	−7.9	−7.1

## Discussion

**FIGURE 9:**
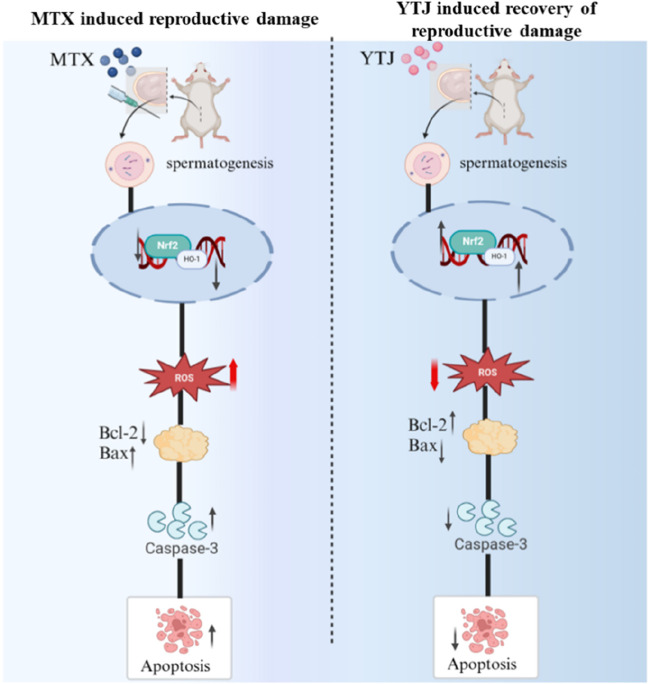
The mechanism of YJT in ameliorating reproductive damage induced by MTX.

Reproductive damage of MTX has been a subject of concern, it’s urgent to find interventions to alleviate toxic and side effect. YJT is one of the effective prescriptions for the treatment of active RA (Gutierrez and Hwang, 2017; Yuan, 2022), and expected to alleviate reproductive toxicity (Lou et al., 2022). In this study, YJT alleviated reproductive damage *in vivo* and reversed OS-induced GC2spd cells apoptosis by regulating Nrf2/HO-1 signaling pathway (Figure 9).

MTX easily causes the death of germ cells, including spermatogonia and spermatocytes. Some studies have reported that (Sinha Hikim and Swerdloff, 1999) MTX-induced reproductive damage was mainly focused on GC2spds (spermatocytes) rather than other spermatogenic cells. Beltrán-Frutos found that (Sinha Hikim and Swerdloff, 1999; Beltrán-Frutos et al., 2022) apoptosis primarily occurs in spermatocytes, whereas spermatogonia are less susceptible. Therefore, the present study mainly targeted spermatocytes to study MTX-induced reproductive injury and a model of MTX-induced GC2 cell injury was constructed. In this study, we demonstrated that MTX significantly diminishes the viability of GC2 cells. To delineate the underlying mechanism of cell death, we employed various death inhibitors and observed that apoptosis inhibitor Z-VAD-FMK effectively rescued GC2spd cells death, indicating that apoptosis is the predominant mode of cell death in spermatogenic cells following MTX exposure. This result was consistent with previous findings. Sukhotnik found (Sukhotnik et al., 2013) that intraperitoneal injection of 20 μg/kg MTX induced germ cell apoptosis and impaired spermatogenesis in rats after 48 h. Based on this point, we assessed apoptosis-related markers and found that YJT treatment reduced the number of TUNEL-positive cells in MTX-treated rats, especially in spermatogonia, spermatocytes and spermatids. Moreover, In addition, YJT increased the expression levels of Bcl2 in MTX-induced testicular tissue and GC2 cells, and decreased the expression levels of Bax and cleaved Caspase 3, which are involved in the apoptotic pathway. Interestingly, we found that MTX-induced reproductive damage was mainly produced through apoptosis, which is similar to the findings of Tousson E (Tousson et al., 2016).

Oxidative damage is a crucial mechanism underlying testicular injury induced by MTX (Bedoui et al., 2019; Abdelhameed et al., 2022). Recent studies have reported that oxidative damage may play a critical role in MTX-reduced nerve, liver and reproductive system damage (Wani et al., 2023; de Almeida Gonçalves et al., 2015). Manna K (Manna et al., 2023) demonstrated that excessive MTX administration to animals leads to lipid peroxidation and the generation of ROS, ultimately leading to liver damage. Hussein O E (Hussein et al., 2020) also demonstrated that prolonged exposure to high levels of MTX can induce ROS-mediated inflammation, cell apoptosis and OS in the liver. MTX can lead to the accumulation of ROS in testicular tissue, which can hinder the activity of vital antioxidant enzymes like SOD and CAT (Martin-Hidalgo et al., 2019; Lopes et al., 2021). OS has the potential to cause apoptosis and significantly worsen testicular toxicity. Although the precise role of OS in MTX-induced sperm apoptosis is not yet fully understood, our study integrated *in vitro* and *in vivo* experiments to investigate the protective mechanism of YJT during the primary development of spermatogonia. Our results showed that YJT decrease the high expression of ROS and increased the expression of SOD, GSH and CAT in rat testicular tissue and GC2spd cells. NAC, which served as a positive control group, showed similar therapeutic effects. Therefore, we concluded that oxidative damage-induced apoptosis mainly occurs at the level of spermatogenic cells and that oxidative damage is the initiating event of spermatogenic apoptosis.

Nrf2 is a vital transcription factor that helps cells to response to OS and maintain a balance of redox reactions within the cell (Tahir et al., 2022). Upon exposure to OS and other triggers, downstream genes like HO-1 are activated at the transcriptional and expression levels, enhancing cellular antioxidant defenses (Tonelli et al., 2018). Increased Nrf2/HO-1 expression enhances both the cellular antioxidant and anti-apoptotic activities, while decreased Nrf2 expression promotes apoptosis (Yang et al., 2020; Abdel-Wahab et al., 2020). The Nrf2/HO-1 pathway is considered a crucial part of the antioxidant defense system, with higher HO-1 expression protecting cells from damage and apoptosis (Biswas et al., 2014). Our studies found that YJT activated Nrf2, which in turn increases HO-1 expression, restoring a balance between pro-apoptotic and anti-apoptotic molecules and preventing excessive spermatogonia apoptosis.

Moreover, our molecular docking results confirm strong binding between YJT’s core components Quercetin, Hyperoside, and Cynaroside and predicted key targets Nrf2 and HO-1 further supporting our findings. Emodin has been demonstrated to prevent severe acute pancreatitis-associated acute lung injury by upregulating the expression of Nrf2 and HO-1 in a dose-dependent manner, as evidenced in both animal models and cell cultures. Molecular docking assays further confirmed the strong binding affinity of Emodin to Nrf2 and HO-1, highlighting its role in activating the Nrf2/HO-1/GPX4 signaling pathway and inhibiting ferroptosis (Shen et al., 2025). Similarly, Hyperoside induces ferroptosis in chronic myeloid leukemia cells by targeting the Nrf2/SLC7A11/GPX4 axis, with molecular docking, DARTS, and CETSA experiments demonstrating its high affinity for Nrf2 (Wei et al., 2024). Additionally, Quercetin has been reported as a promising therapeutic agent for intervertebral disc degeneration, potentially through binding to the Keap1-Nrf2 complex and suppressing the NF-κB pathway (Shao et al., 2021). These findings collectively support the critical role of Nrf2/HO-1 activation in mediating the protective effects of these compounds.

Our study pioneered a novel approach by integrating both *in vitro* and *in vivo* experimental models to delineate the mechanisms underlying MTX-induced reproductive dysfunction. Our study specifically focused on spermatogenic cells and demonstrated that apoptosis is the predominant mode of cell death in spermatogenic cells following MTX exposure. Subsequent researchers have revealed that oxidative stress triggers apoptosis, and this process is upon the activation of the Nrf2/HO-1 signaling pathway. These findings not only deepened our understanding of the pathological processes induced by MTX but also identified a novel therapeutic target for ameliorating the toxic and side effect of MTX on reproductive function. Additionally, our findings demonstrated that the Chinese herbal formula YJT can effectively mitigate the toxic and side effect of MTX on spermatogenic cells, thereby enhancing fertility. This underscored the potential of Chinese pharmacopeia in modern medical practice, providing a novel perspective on the complementary nature of Eastern and Western medical approaches. While these results are promising, it is important to note that the mechanisms and efficacy observed in preclinical models require further validation in human studies. We acknowledge that the current findings on YJT are primarily based on animal and cellular models, which may not fully reflect its effects in humans. In future studies, we aim to validate the efficacy and safety of YJT through clinical trials and evidence-based medical research, providing a more comprehensive understanding of its therapeutic potential.

## Conclusion

In conclusion, our study suggested that YJT treatment reduced MTX-induced reproductive toxicity in the male rat reproductive system. This protection was achieved by modulating OS-induced cell apoptosis through the Nrf2/HO-1 signaling pathway. YJT restored the balance between pro-apoptotic and anti-apoptotic molecules, reduced oxidative damage, and protected normal spermatogenesis.

## Data Availability

The original contributions presented in the study are included in the article/Supplementary Material, further inquiries can be directed to the corresponding authors.

## Abbreviations

MTX, Methotrexate; RA, rheumatoid arthritis; YJT, Yunpi Jiedu Tongluo Qushi Decoction; UPLC-MS, Ultra Performance Liquid Chromatography-Mass Spectrometry; OS, Oxidative Stress; ROS, Reactive Oxygen Species; TCM, Traditional Chinese medicine; T, Testosterone; LH, Luteinizing Hormone; FSH, Follicle-Stimulating Hormone; Nrf2, Nuclear factor E2-related factor 2; Bax, BCL2-associated X protein; Bcl2, B-cell lymphoma-2; SOD, Superoxide Dismutase; CAT, Catalase; HO-1, Heme oxygenase-1; GAPDH, Glyceraldehyde-3-phosphate dehydrogenase.
