# Supplementary material for: Exploring the protective mechanisms of the yunpi jiedu tongluo qushi decoction on methotrexate-induced reproductive damage in male rats based on Nrf2/HO-1 signaling pathway
Source: Front Cell Dev Biol. 2025 Aug 22;13:1626955. doi: 10.3389/fcell.2025.1626955 (PMC12411506; doi:10.3389/fcell.2025.1626955)

**Supplementary Materials**

**Figure S1 Qualitative analysis of nine major bioactive components in the YJT by UPLC-MS**

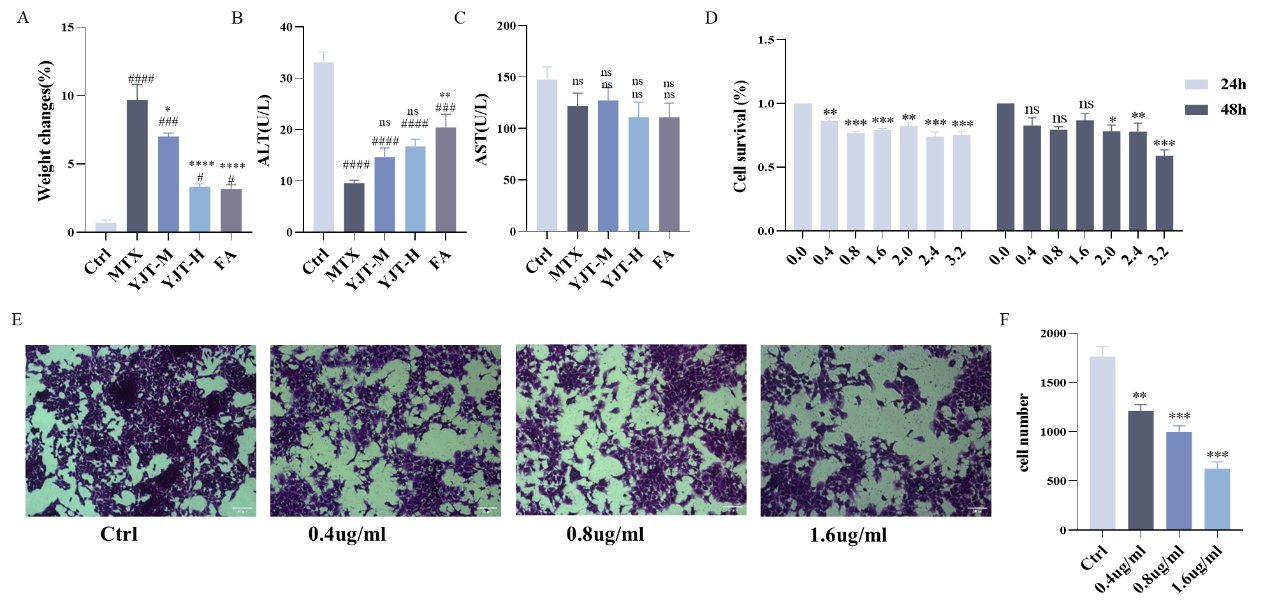


**Figure S2 Safety evaluation in rats and effect of MTX on GC2spd cells.**

(A)Effects of YJT on the changes in body weight induced by MTX in rats. (n=10) (B, C) Changes in liver function in various rat groups treated with YJT and MTX(n=6-10). (D) Cytotoxicity assay of MTX(n=6); (E-F) Crystalline violet staining to detect the effect of MTX on the proliferation of GC2spd cells. Data are presented as Mean ± SEM. # compared to the control group; * compared to the MTX group; ns indicates no significant difference, # *P*<0.05; ## *P*<0.01; ### *P*<0.001; * *P*<0.05; ** *P*<0.01; *** *P*<0.001.


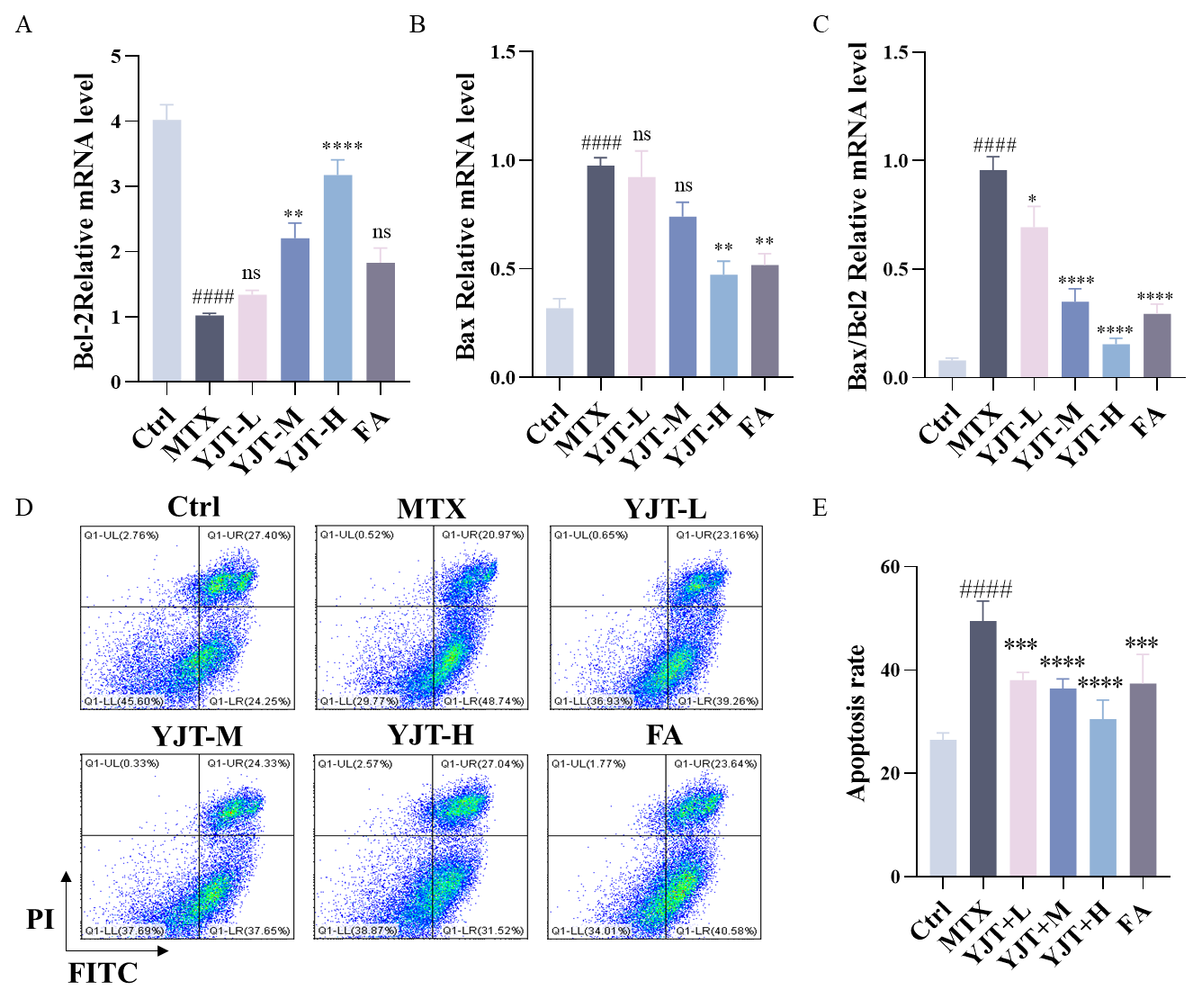


**Figure S3 Effect of YJT on apoptosis in MTX-intervened GC2spd cells.**

(A-C) mRNA expression levels of Bcl2 (A), Bax (B), and Bax/Bcl2 (C) in GC2spd cells(n=9). (D-E) YJT improves MTX-induced apoptosis in GC2spd cells(n=3). Data are presented as Mean ± SEM. # compared to the control group; * compared to the MTX group; ns indicates no significant difference, # *P*<0.05; ## *P*<0.01; ### *P*<0.001; * *P*<0.05; ** *P*<0.01; *** *P*<0.001.


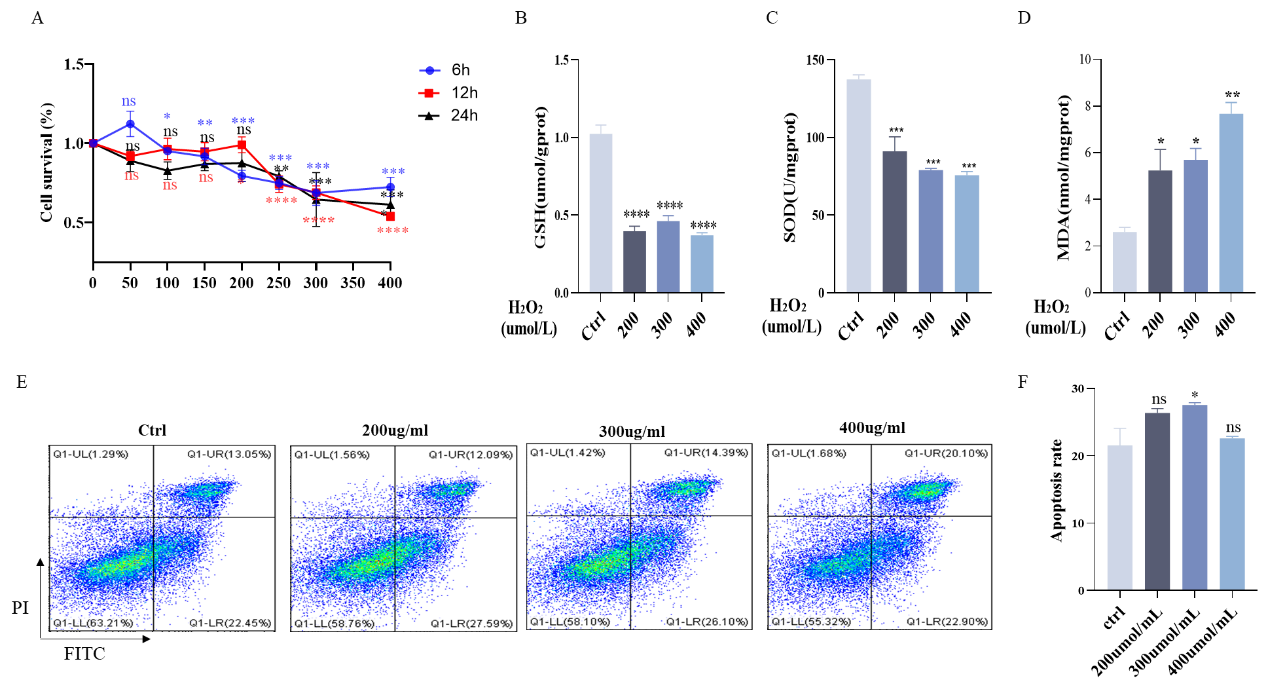


**Figure S4 A cellular model of H_2_O_2_-induced oxidative damage**.

(A) Detection of cytotoxicity of H_2_O_2_ on GC2spd cells(n=6). (B-D) Colorimetric assay to detect the content of GSH,SOD,and MDA in GC2spd cells interfered by H_2_O_2_(n=6). (C) Flow cytometry to detect the effect of different concentrations of H_2_O_2_ on apoptosis of GC2spd cells(n=3).


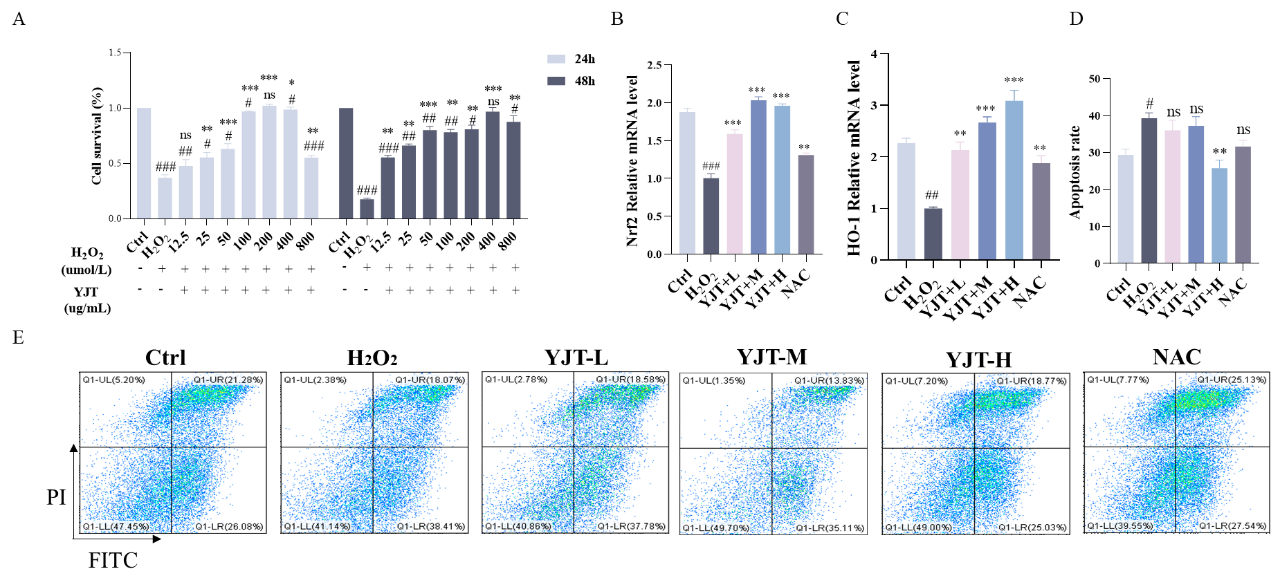


**Figure S5 Detection of apoptosis and related signaling pathway proteins in cell models of oxidative damage.**

1. CCK8 detects the toxicity of YJT in oxidative damage cell model(n=6). (B-C) qRT-PCR detection of mRNA expression of Nrf2, HO-1 in oxidative damage cell model(n=9). (D-E) Flow cytometry to detect apoptosis in oxidative damage cell model(n=3).

**Figure S6** **Molecular docking results for folic acid, NRF2, and HO-1 activators.**


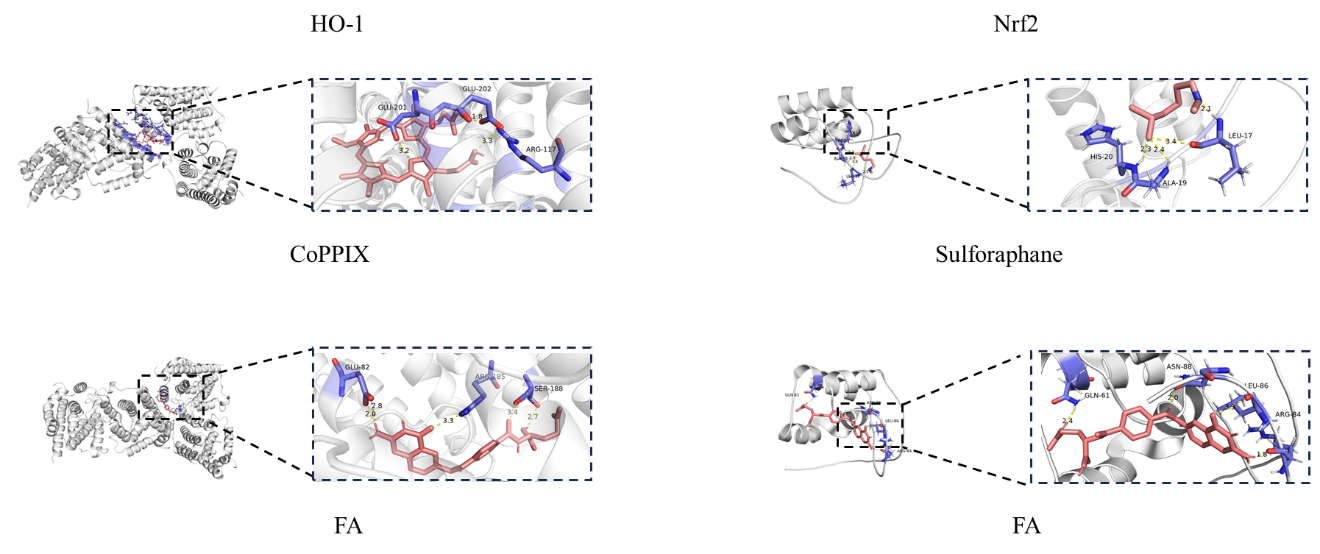


**Figure S7 Protein Database (PDB) IDs of proteins and PubChem compound IDs (CIDs) of ligands used in docking studies.**


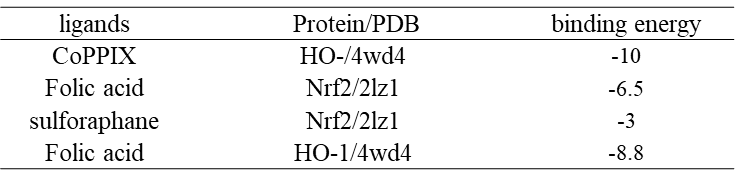


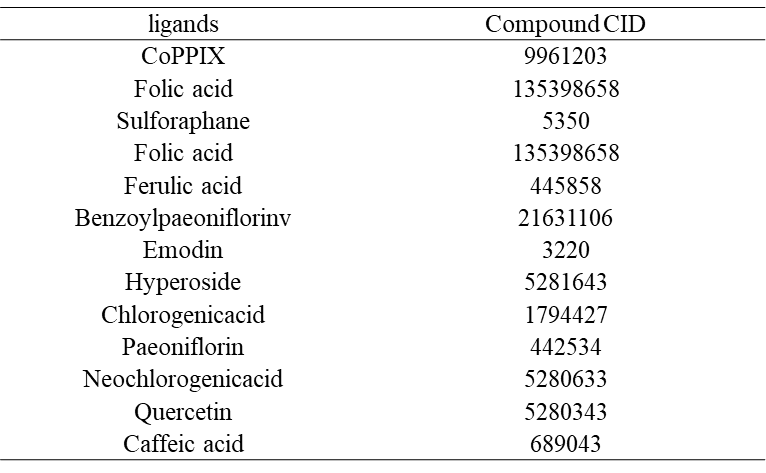

Supplement: Supplementary file 1 [file DataSheet1.docx]
